# Predicting future surgical steps during MCA aneurysm clipping using a multimodal transformer

**DOI:** 10.3389/fsurg.2026.1827725

**Published:** 2026-06-02

**Authors:** Thomas J. On, Jonathan A. Tangsrivimol, Jiuxu Chen, Yuan Xu, Baoxin Li, Michael T. Lawton, Mark C. Preul

**Affiliations:** 1 The Loyal and Edith Davis Neurosurgical Research Laboratory, Department of Neurosurgery, Barrow Neurological Institute, St. Joseph's Hospital and Medical Center, Phoenix, Arizona; 2 Division of Neurosurgery, Department of Surgery, Chulabhorn Hospital, Chulabhorn Royal Academy, Bangkok, Thailand; 3 School of Computing and Augmented Intelligence, Arizona State University, Tempe, Arizona

**Keywords:** anticipating surgical steps, artificial intelligence, middle cerebral artery aneurysm, neurosurgical education, predicting surgical steps, surgical video, visual-annotation model

## Abstract

**Objective:**

Intracranial aneurysm clipping is technically demanding, with dynamic anatomy and evolving intraoperative decisions. Although AI has been applied to retrospective surgical phase recognition, near-future surgical step prediction remains largely unexplored in neurosurgery. This study evaluated the feasibility of fixed-horizon surgical step prediction in recorded microscope videos of middle cerebral artery aneurysm clipping operations.

**Methods:**

We retrospectively analyzed 25 uncomplicated MCA bifurcation aneurysm clipping surgeries by a single neurosurgeon, using 18 for training and 7 for independent testing. Cases were annotated into 12 standardized operative steps. A transformer-based prediction framework was evaluated with three input configurations: video features alone, prior human-annotated step labels alone, and combined video plus step-label inputs. With a sliding-window approach, each configuration used 1 min of input data to predict the operative step labels occurring during the subsequent 1 min. Performance was assessed by accuracy, weighted F1 score, and sequence-level agreement with ground truth.

**Results:**

The multimodal model achieved the highest mean accuracy and weighted F1 score, 0.683 and 0.673, compared with 0.606 and 0.577 for the annotation-only model and 0.477 and 0.447 for the video-only model. The multimodal model also showed the best sequence-level alignment, with a normalized edit distance of 0.430 and edit score of 0.570.

**Conclusion:**

Fixed-horizon surgical step prediction during MCA aneurysm clipping was feasible under controlled input conditions. Multimodal modeling provided the strongest predictive performance. These findings represent upper-bound performance and require validation in fully automated recognition-to-prediction pipelines.

## 1 Introduction

Intracranial aneurysm clipping is a complex neurosurgical procedure characterized by variable anatomy, unpredictable intraoperative findings, and dynamic sequencing of surgical steps. Accurate anticipation of subsequent surgical actions is essential for operative efficiency, safety, and effective trainee education (1, 2). Among intracranial aneurysms, the surgical treatment of middle cerebral artery (MCA) aneurysms is especially suitable for detailed analysis due to their frequent surgical indication, anatomical variability, and well-documented surgical approaches (3–5).

Artificial intelligence (AI)-driven approaches have increasingly been used to enhance surgical decision-making, procedural efficiency, and educational outcomes across various surgical fields (6, 7). Previous studies mainly focused on recognizing surgical steps with convolutional neural network (CNN) architectures combined with recurrent neural networks, especially long short-term memory (LSTM) models (8–10). However, these models primarily target surgical step recognition rather than anticipation, which limits their practical use in real-time clinical settings.

In computer vision, human activity analysis has progressed from recognizing ongoing actions to predicting future events, a capability relevant to applications such as assistive robotics and intelligent surveillance systems (11). Although substantial advances have been made in action forecasting within structured, non-medical domains, translation of these methods to surgery remains limited (12–14). Transformer-based models, initially developed in natural language processing, have shown strong performance in modeling sequential dependencies and contextual relationships, making them well suited for complex, dynamic surgical workflows (15). However, their use for anticipating future operative steps in neurosurgery, including MCA aneurysm clipping, has not been explored.

We previously used a CNN-LSTM automated approach to recognize surgical phases in MCA aneurysm clipping (10). In the present study, we evaluated transformer-based models for near-future surgical step prediction using a sliding fixed-horizon framework. Models predicted the surgical step or sequence of steps occurring over the subsequent 60 s using encoded microscope video features, preceding human-annotated ground-truth step labels, or both. By comparing video-only, annotation-only, and multimodal models, we sought to determine whether near-future surgical step prediction is feasible under controlled input conditions and whether visual features and prior procedural context provide complementary predictive information.

## 2 Methods

### 2.1 Data acquisition and case selection

We reviewed 42 videos of MCA aneurysm clipping procedures recorded with an operative microscope, performed by a single neurosurgeon (M.T.L.) at our institution from October 2020 to October 2024. Videos were sourced from a de-identified institutional neurosurgical operative video repository, and because the operative feeds contained no patient-identifying information, this study was exempt from Institutional Review Board review.

To reduce procedural variability, we selected 25 uncomplicated cases of single MCA bifurcation aneurysms. Cases with multiple aneurysms, bypass procedures, giant aneurysms requiring complex clip reconstruction, recurrent aneurysms, or incomplete data were excluded. The final cohort was randomly divided into a training set of 18 cases and an independent testing set of 7 cases.

### 2.2 Surgical step annotation

Videos captured only the part of each operation performed by the attending neurosurgeon. Preparatory steps done by residents were excluded. Cases were annotated into 12 standardized surgical steps using established neurosurgical workflow definitions and operative notes (2, 16–18). These included sylvian fissure splitting, M4-M2 branch dissection, M1 segment preparation, supraclinoid ICA dissection, A1 anterior cerebral artery dissection, aneurysm neck dissection, aneurysm mobilization, proximal control, clip application, occlusion confirmation, release of proximal control, and aneurysmectomy. Indocyanine green (ICG) video angiography was labeled as a separate workflow label. Annotations were independently performed by two neurosurgeons (Y.X., J.A.T.), with cases divided between reviewers rather than annotated in duplicate.

### 2.3 Video preprocessing

Microscope videos recorded at 30 frames per second with a resolution of 1,920 × 1,080 pixels were downsampled to one representative frame per second by extracting the center frame of each second. Frames were center-cropped to 1,080 × 1,080 pixels, resized to 224 × 224 pixels, and normalized to facilitate model training and computational efficiency.

### 2.4 Model architecture

**Figure 1:**
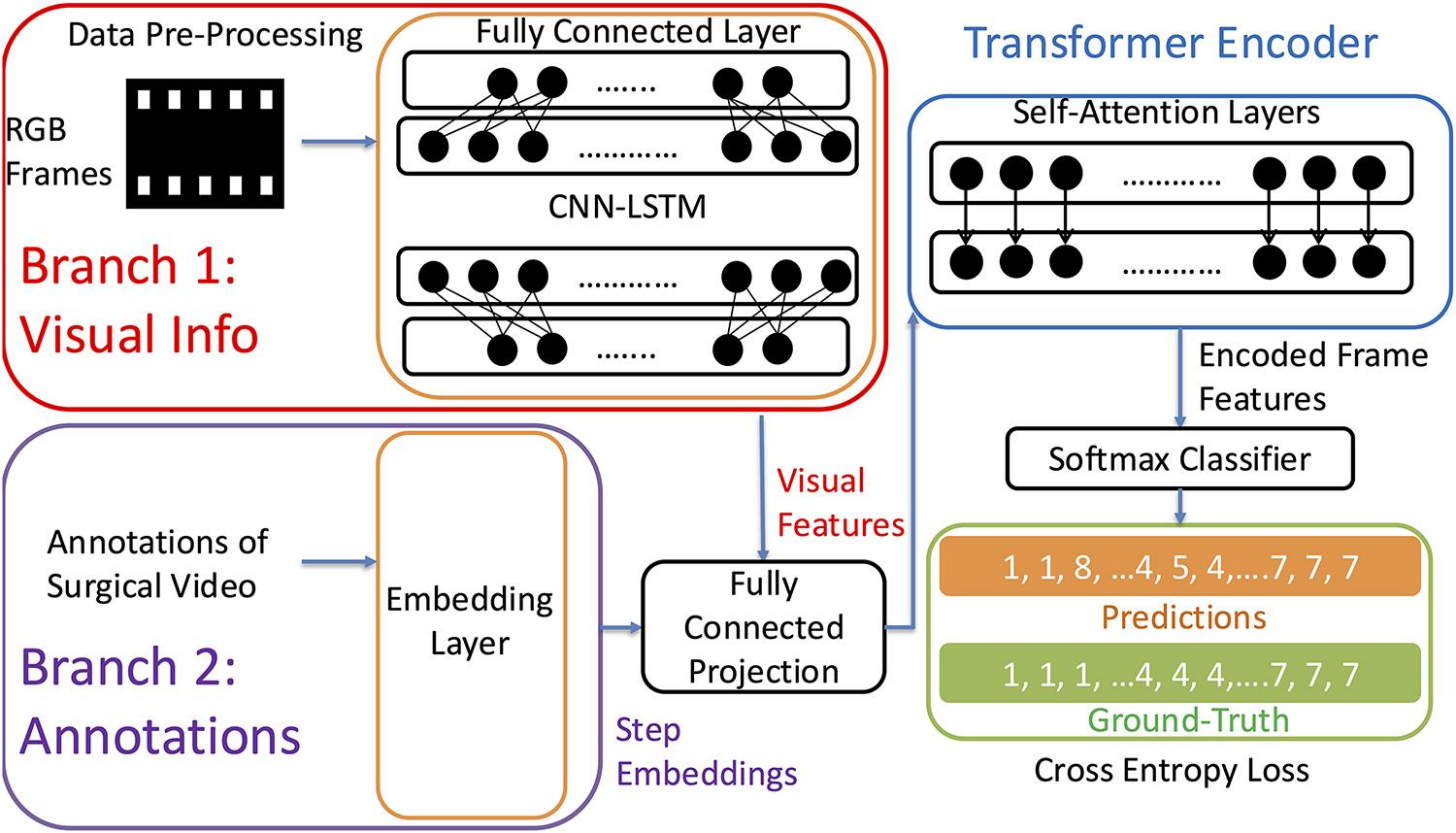
Multimodal transformer architecture for future surgical step prediction. RGB microscope frames were processed through a CNN-LSTM visual branch, and observed ground-truth surgical step labels were encoded through an annotation branch. The resulting features were concatenated, projected through a fully connected layer, processed by a transformer encoder with multi-head self-attention, and passed to a softmax classifier to predict future surgical step labels.

A transformer-based neural network was developed to predict future surgical step labels from observed operative data. The multimodal architecture is illustrated in Figure 1. Three input configurations were evaluated: visual-only, annotation-only, and multimodal.

In the visual-only model, RGB video frames were processed using a previously trained CNN-LSTM architecture to extract frame-level visual features (10). After removal of the final classification layer, the resulting 256-dimensional feature vectors were passed to a transformer encoder to predict future surgical step labels.

In the annotation-only model, ground-truth surgical step labels from the observed portion of the case were converted into 256-dimensional embeddings using a learned embedding layer and then processed by the transformer encoder to predict future surgical step labels based on procedural context alone.

In the multimodal model, visual embeddings from the CNN-LSTM and embeddings of ground-truth surgical step labels were concatenated into a 512-dimensional vector, projected to 256 dimensions through a fully connected layer, and then processed by the transformer encoder to generate predictions. All three models used a two-layer transformer encoder with multi-head self-attention and a softmax classifier to output predicted surgical step labels.

### 2.5 Prediction task definition

Models used a 60-s sliding input window consisting of visual features, preceding ground-truth workflow labels, or both. For each input window, the model predicted workflow labels over the subsequent fixed 60 s horizon across the duration of each case.

### 2.6 Training

Models were trained using supervised learning on the 18-case training set. Cross-entropy loss was optimized with the Adam optimizer at a learning rate of 0.0001 and a batch size of 16. Dropout at a rate of 0.3 and early stopping were used to prevent overfitting.

### 2.7 Evaluation metrics

Performance was assessed on the independent testing set using frame-level predictions within the future 60 s prediction window. To summarize case-level performance, frame-level accuracy and weighted F1 score were calculated for each test case. Accuracy was defined as the proportion of correctly classified frames. Weighted F1 score was calculated by determining the F1 score for each surgical step and then averaging these scores using the number of ground-truth frames in each step as weights, making the final score proportional to step frequency within the case.

To summarize pooled step-level performance across the testing cohort, precision, recall, and F1 score were calculated for each surgical step. Precision reflected the proportion of predicted instances that were correct, recall reflected the proportion of true instances detected, and F1 score provided their harmonic mean. All metrics were presented separately for the visual-only, annotation-only, and multimodal models.

### 2.8 Sequence-level workflow analysis

We assessed overall sequence error for each case by comparing the predicted step sequence with the true operative sequence using Levenshtein edit distance, which counts the minimum number of step insertions, deletions, and substitutions required to transform the predicted sequence into the reference sequence. To account for variability in operative duration, edit distance was normalized by the length of the ground-truth sequence. We also calculated an edit score defined as 1 minus the normalized edit distance, so that a lower normalized distance and a higher edit score indicate better structural alignment. Sequence-level metrics were averaged across all testing cases and reported separately for the visual-only, annotation-only, and multimodal model. This analysis assessed the preservation of global temporal structure and stage transition order.

## 3 Results

### 3.1 Dataset characteristics

The final dataset consisted of 25 operative microscope video cases, with 18 cases assigned to the training set and 7 cases reserved for independent testing. In the independent testing cohort, 4 aneurysms were right-sided and 3 were left-sided. Aneurysm projection varied across cases and included lateral, inferior, superior, and anterior orientations. Proximal control was used in 4 of 7 cases, and ICG videoangiography was used in all cases. Detailed case-level characteristics of the independent testing cohort are provided in Table 1. Across all annotated videos, the dataset included 27,180 training frames and 10,620 testing frames. Table 2 shows the distribution of annotated frames across surgical steps in the training and testing cohorts.

**Table 1:** Case-level characteristics of the independent testing cohort.

Case	Side	MCA location	Aneurysm projection	Proximal control used	ICG used
Case 1	Left	MCA bifurcation	lateral	yes	yes
Case 2	Left	MCA bifurcation	inferior	no	yes
Case 3	Right	MCA bifurcation	superior	yes	yes
Case 4	Left	MCA bifurcation	superior	no	yes
Case 5	Right	MCA bifurcation	inferior	yes	yes
Case 6	Right	MCA bifurcation	anterior	no	yes
Case 7	Right	MCA bifurcation	lateral	yes	yes

**Table 2:** Distribution of annotated frames across surgical steps in the training and testing cohorts.

Video annotation category	Training frames, *n*	Testing frames, *n*
Sylvian fissure splitting (step 1)	9,402	3,420
M4 segment to M2 branch dissection (step 2)	5,683	1,313
M1 segment preparation (step 3)	777	107
Supraclinoid ICA dissection (step 4)	826	52
A1 ACA dissection (step 5)	84	67
Aneurysm neck dissection (step 6)	677	141
Aneurysm mobilization (step 7)	5,916	3,754
Proximal control (step 8)	95	57
Clip application (step 9)	1,957	584
Occlusion confirmation (step 10)	370	260
Release of proximal control (step 11)	52	32
Aneurysm resection (step 12)	69	153
ICG videoangiography	1,272	680
Total	27,180	10,620

ACA, anterior cerebral artery; ICA, internal carotid artery; ICG, indocyanine green.

### 3.2 Distribution of 60 s prediction windows

To characterize the prediction target, we quantified whether each 60 s prediction interval remained within the same surgical step or crossed at least one surgical step boundary. Overall, 45.2% of 60 s prediction intervals crossed at least one surgical step boundary, whereas 54.8% remained within a single surgical step. Among the boundary-crossing intervals, 27.5% required prediction across three or more distinct surgical phases within the 60 s horizon.

### 3.3 Model performance

Case-level performance in the independent testing cohort is summarized in Table 3 using frame-level accuracy and weighted F1 score. The multimodal model showed the strongest performance across all 7 test cases, with a case-level mean accuracy of 0.683 and weighted F1 of 0.673, compared with 0.606 and 0.577 for the annotation-only model and 0.477 and 0.447 for the visual-only model. A subset analysis was performed to evaluate model performance during fixed 1 min prediction horizons that crossed surgical step boundaries (Table 4). In prediction horizons crossing exactly 2 surgical steps, the multimodal model achieved an accuracy of 0.509 and weighted F1 of 0.516. In prediction horizons crossing 3 or more surgical steps, the multimodal model achieved an accuracy of 0.420 and weighted F1 of 0.372.

**Table 3:** Overall case-level model performance in the independent testing cohort.

Case	Visual-only accuracy	Visual-only weighted F1	Annotation-only accuracy	Annotation-only weighted F1	Multimodal accuracy	Multimodal weighted F1
Case 1	0.607	0.603	0.635	0.615	0.658	0.643
Case 2	0.485	0.396	0.633	0.601	0.711	0.706
Case 3	0.327	0.337	0.749	0.741	0.791	0.783
Case 4	0.526	0.497	0.535	0.492	0.621	0.616
Case 5	0.362	0.304	0.432	0.413	0.538	0.529
Case 6	0.537	0.503	0.625	0.555	0.694	0.684
Case 7	0.495	0.491	0.633	0.624	0.766	0.748
Case-level mean	0.477	0.447	0.606	0.577	0.683	0.673

**Table 4:** Model performance in boundary-crossing prediction horizons.

Subset	Model	Accuracy	Weighted F1
Exactly 2 surgical phases crossed	Visual-only	0.425	0.428
Exactly 2 surgical phases crossed	Annotation-only	0.481	0.477
Exactly 2 surgical phases crossed	Multimodal	0.509	0.516
3 or more surgical phases crossed	Visual-only	0.202	0.180
3 or more surgical phases crossed	Annotation-only	0.385	0.341
3 or more surgical phases crossed	Multimodal	0.420	0.372

Step-specific precision, recall, and F1 scores are reported in Table 5. Performance varied substantially across surgical steps. The annotation-only model showed strong performance for longer and more temporally structured steps, such as sylvian fissure splitting and aneurysm mobilization, with F1 scores of 0.823 and 0.787, respectively. However, it also showed several zero or undefined values for shorter steps, indicating limited detection of these classes. The multimodal model showed more balanced performance across steps, with strong F1 scores for sylvian fissure splitting at 0.852, M4 segment to M2 branch dissection at 0.545, and aneurysm mobilization at 0.812. The visual-only model frequently showed high precision but low recall, suggesting that predictions were often correct when made, although many true instances were missed.

**Table 5:** Class-wise precision, recall, and F1 score for surgical step anticipation across input modalities.

Surgical step	Visual-only precision	Visual-only recall	Visual-only F1	Annotation-only precision	Annotation-only recall	Annotation-only F1	Multimodal precision	Multimodal recall	Multimodal F1
Sylvian fissure splitting (step 1)	0.577	0.368	0.449	0.799	0.848	0.823	0.834	0.871	0.852
M4 segment to M2 branch dissection (step 2)	0.274	0.264	0.269	0.409	0.439	0.424	0.514	0.580	0.545
M1 segment preparation (step 3)	1.000	0.168	0.288	0.000	0.000	–	0.333	0.243	0.281
Supraclinoid ICA dissection (step 4)	1.000	0.154	0.267	0.000	0.000	–	0.308	0.154	0.205
A1 ACA dissection (step 5)	1.000	0.209	0.346	–	0.000	–	1.000	0.209	0.346
Aneurysm neck dissection (step 6)	1.000	0.270	0.425	–	0.000	–	1.000	0.305	0.467
Aneurysm mobilization (step 7)	0.419	0.769	0.542	0.745	0.834	0.787	0.791	0.834	0.812
Proximal control (step 8)	1.000	0.140	0.246	–	0.000	–	1.000	0.123	0.219
Clip application (step 9)	0.892	0.142	0.245	0.210	0.214	0.212	0.283	0.330	0.305
Occlusion confirmation (step 10)	1.000	0.100	0.182	–	0.000	–	1.000	0.173	0.295
Release of proximal control (step 11)	–	0.000	–	–	0.000	–	–	0.000	–
Aneurysm resection (step 12)	1.000	0.157	0.271	–	0.000	–	1.000	0.216	0.355
ICG videoangiography	0.143	0.010	0.019	0.577	0.625	0.600	0.643	0.638	0.641

Dashes indicate undefined values due to zero predicted instances for that class.

**Figure 2:**
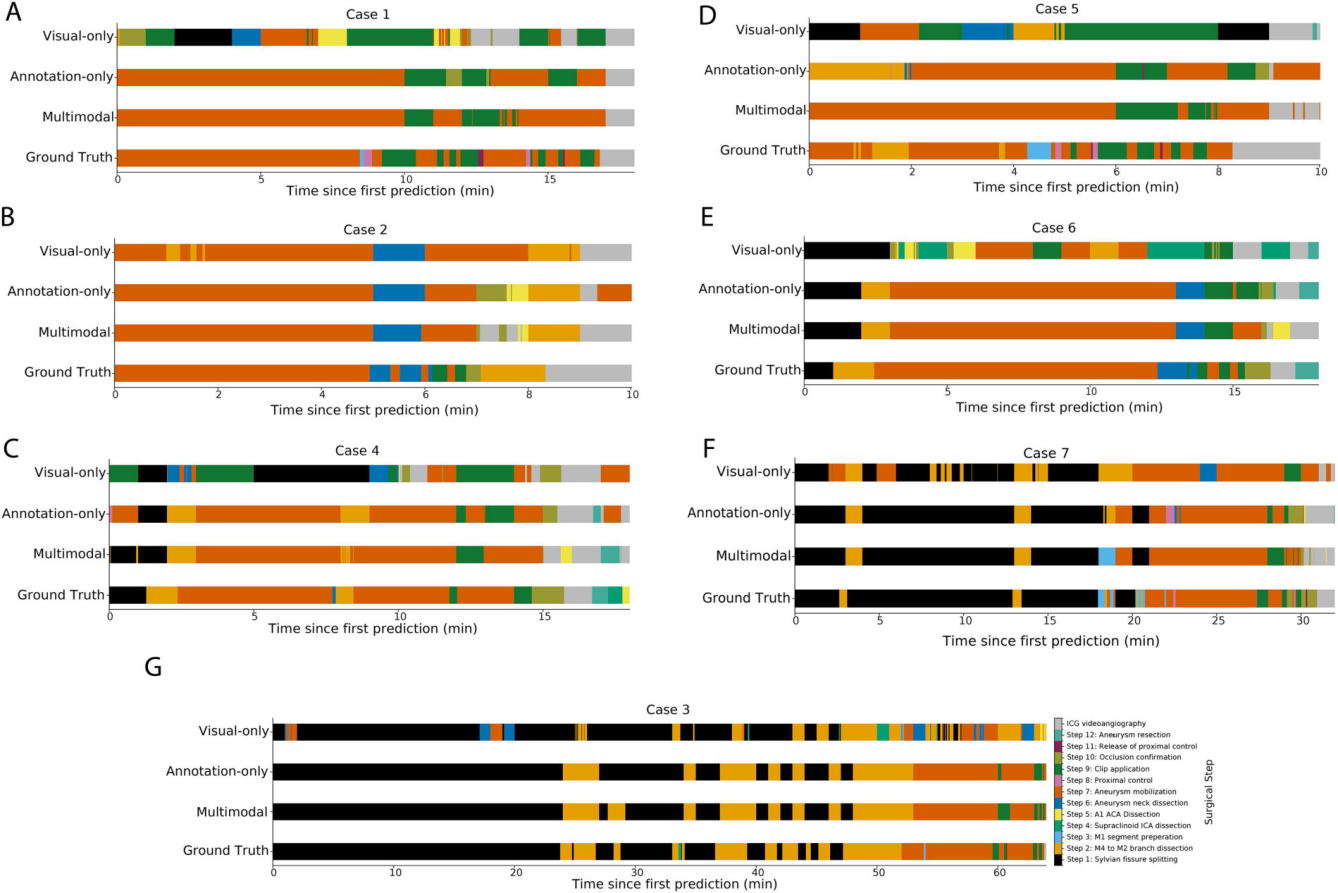
Temporal alignment of predicted and ground-truth surgical step sequences across test cases. Panels **A–C** correspond to Cases 1, 2, and 4; panels **D–F** correspond to Cases 5, 6, and 7; and panel **G** corresponds to Case 3. Each panel represents one case, with rows showing video-only, annotation-only, and multimodal model predictions alongside the ground-truth sequence. Colors indicate distinct surgical steps, enabling comparison of predicted step timing, transitions, and overall sequence agreement across model configurations.

Analysis of the predicted step order further demonstrated the advantage of multimodal integration (Figure 2). The visual-only model showed poor agreement with the true operative sequence, with a normalized edit distance of 0.855 (edit score 0.145). Adding ground-truth step labels substantially improved sequence consistency, reducing the normalized edit distance to 0.456 (edit score 0.544). The multimodal model achieved the best temporal alignment, with a normalized edit distance of 0.430 (edit score 0.570), indicating better preservation of the overall step sequence.

## 4 Discussion

This study presents a transformer-based framework for fixed-horizon surgical step prediction during MCA aneurysm clipping. It should be interpreted as an early feasibility evaluation in a controlled experimental setting, rather than as a complete real-time deployment system. Using a sliding 60 s horizon, the models predicted the surgical step or sequence of steps expected in the subsequent minute from encoded video features, preceding human-annotated step labels, or both. The use of ground-truth labels provided a controlled best-case estimate of how accurately near-future surgical progression could be predicted when prior accurate procedural context was known.

In comparing the three model configurations, prior step-label context appeared most useful for longer, temporally structured steps, such as sylvian fissure splitting and aneurysm mobilization, whereas encoded video features appeared to contribute more during anatomically or technically defined steps, including aneurysm neck dissection and clip-related maneuvers. The stronger performance of the multimodal model suggested that integrating these inputs allowed the model to capture both broad operative progression and local visual cues. Sequence-level analysis further showed that the multimodal model had the closest agreement with the true operative step order by edit distance, suggesting better preservation of overall surgical progression beyond individual step-label prediction.

Prediction performance also depended on the structure of the 60 s horizon itself. When attempting to predict the next minute of surgery, the task is not always the same. In some intervals, the correct prediction is that the current surgical step will continue. In others, the model must anticipate that the operation will move into a different step or even pass through multiple steps within the same 60 s horizon. These scenarios may differ in difficulty. Predicting step continuation may depend largely on recognizing procedural stability, whereas predicting step changes may require identifying cues that the operative workflow is changing. In this study, nearly half of the prediction horizons crossed at least one step boundary. Performance decreased modestly in horizons crossing exactly two surgical steps and decreased more noticeably in horizons crossing three or more surgical steps, suggesting that prediction becomes increasingly difficult as the number of step transitions within the prediction horizon increases.

Most previous work in surgical AI has focused on retrospective recognition of surgical phases. Recognition systems identify what is happening rather than what will happen next (6–8, 10). Action anticipation has been extensively studied in computer vision, where models forecast future events in dynamic visual sequences, but this approach has not been applied to neurosurgical workflows before (11–14). This work provides an early framework for studying surgical AI systems that go beyond retrospective step recognition. However, translation toward clinical use would require a fully automated recognition-to-prediction pipeline, in which the current surgical step is first identified from video and then used, together with visual features, to predict upcoming steps. In such a pipeline, recognition errors could propagate into downstream prediction and reduce performance. Predictive modeling may nevertheless help characterize surgical progress and estimate where the operation is heading, rather than simply identifying the current visible step.

Beyond model development, the feasibility of surgical AI in neurosurgery depends on overcoming substantial logistical and technical barriers to data generation. Patients must consent to operative video recording, operating microscopes must be equipped for high-quality capture, video quality must be verified, and the resulting data must be securely stored and anonymized. Because operative videos are large, scalable storage infrastructure is also required. Each case must then be reviewed and annotated by individuals with neurosurgical expertise, making dataset creation time-intensive and difficult to scale. These barriers are particularly relevant in cerebrovascular neurosurgery, where procedures are less standardized than many laparoscopic workflows and where more complex aneurysms or cases from other vascular territories would likely introduce additional variability.

### 4.1 Limitations

This study has several limitations and considerations. All procedures were performed at a single institution by one neurosurgeon, and the independent testing cohort was small. These factors may limit generalizability and should be addressed in larger multicenter, multi-surgeon studies.

Manual labeling may introduce variability in ground-truth definitions. In this study, annotations were performed by two neurosurgeons. However, cases were divided between reviewers rather than annotated in duplicate, precluding formal assessment of inter-rater agreement. Although operative steps were clear and distinguishable in this cohort of uncomplicated MCA bifurcation aneurysms, step boundaries may be less consistent in more complex cases. Future studies may need to directly assess inter-rater agreement in those settings.

The 60 s prediction horizon was selected as a clinically reasonable interval for near-future surgical step prediction. This interval was intended to provide useful anticipatory information while remaining close enough to the current operative context to be predictable. However, the threshold is inherently arbitrary, and we did not perform a sensitivity analysis across alternative horizons. Future studies should evaluate multiple time intervals to characterize how performance varies with horizon length and to identify the most clinically useful prediction windows.

## 5 Conclusions

This study demonstrates the feasibility of fixed-horizon surgical step prediction during MCA aneurysm clipping in a controlled experimental setting. The multimodal model showed the strongest performance, suggesting that encoded video features and preceding human-annotated step context provide complementary information for predicting near-future surgical progression. Because prior step labels were manually annotated rather than generated by automated recognition, these findings should be interpreted as upper-bound performance under accurate step-input conditions. Future work should validate this approach in larger, more diverse cohorts and develop fully automated recognition-to-prediction pipelines before clinical implementation.

## Data Availability

The raw data supporting the conclusions of this article will be made available by the authors, without undue reservation.
